# Semi-continuous adsorption-biocatalysis systems using waste-derived biochar functionalized with laccase for diclofenac removal from wastewater

**DOI:** 10.3389/fchem.2026.1814099

**Published:** 2026-05-25

**Authors:** Rita Gouveia, Ângela Almeida, Érika M. L. Sousa, María. V. Gil, Francisco A. da Silva, Vânia Calisto

**Affiliations:** 1 Department of Chemistry, University of Aveiro, Aveiro, Portugal; 2 Department of Chemistry and CESAM, University of Aveiro, Aveiro, Portugal; 3 Instituto de Ciencia y Tecnología del Carbono, (INCAR), CSIC, Oviedo, Spain; 4 Department of Chemistry and CICECO, University of Aveiro, Aveiro, Portugal

**Keywords:** batch system, carbon-based materials, enzymatic immobilization, fixed-bed column, pharmaceutical removal, residual biomass, stirred tank

## Abstract

Diclofenac (DCF) is a widely consumed anti-inflammatory drug, frequently detected in aquatic environments worldwide due to the limited removal efficiency of conventional wastewater treatment processes. This work investigates the valorisation of brewery waste into functionalized carbon-based materials to mitigate the entry of DCF into aquatic systems. Biochar (BC) was produced from spent brewery grains via microwave-assisted pyrolysis, contributing to Sustainable Development Goal 12 by repurposing agro-industrial by-products. A biocatalytic composite (BC-LAC) was synthesized through laccase (*Trametes versicolor*, ≥0.5 U/mg) immobilization onto BC. The materials produced (BC and BC-LAC) were characterized by N_2_ physisorption and scanning electron microscopy, revealing a well-developed microporous structure, with specific surface areas of 301 m^2^ g^-1^ and 191 m^2^ g^-1^ for BC and BC-LAC, respectively. DCF removal from aqueous matrices (buffered ultrapure water and urban wastewater) was evaluated using BC and BC-LAC, under batch conditions and in semi-continuous operation mode, employing stirred tank and fixed-bed column configurations. For the semi-continuous operation modes, the effect of materials dose (0.5–2.0 g L^-1^) and flow rate (6.94 ⋅ $10^{-4}$ – 2.78 ⋅ $10^{-3}$ L min^-1^) in the breakthrough curves were evaluated. In wastewater, fixed-bed column experiments with BC showed higher removal efficiency than BC-LAC, with the former treating a volume of effluent five times higher than the latter. Nevertheless, in the stirred tank, BC-LAC breakthrough curves revealed an initial improvement in DCF removal compared with BC, suggesting enhanced hydrodynamics and oxygen availability. Despite this fact, the treated volume (0.25 and 0.26 L, for BC and BC-LAC, respectively), as well as the operating times, are similar for both materials, under the tested conditions (0.5 g L^-1^ of materials and 6.94 ⋅ $10^{-4}$ L min^-1^). Overall, the results confirm the potential of the produced waste-derived BC as an effective adsorbent, in line with circular strategies and sustainable water treatment. The results highlight that the application of BC-LAC did not present enhanced cost-effectiveness, requiring further optimization to enable a successful integration of combined adsorption and enzymatic degradation approach.

## Highlights

- Brewery spent grain biochar (BC) was produced via microwave-assisted pyrolysis.
- Diclofenac (DCF) removal was assessed in fixed-bed and stirred tank systems.
- Laccase (LAC) immobilized on BC (BC-LAC) was tested to enhance DCF degradation.
- BC showed higher DCF adsorption capacity than BC-LAC, in both systems.
- BC-LAC did not present enhanced cost-effectiveness in the tested conditions.

## 1 Introduction

In recent decades, the growing presence of pharmaceutical compounds in wastewater has become a major environmental concern. Their pseudo-persistent behaviour, resulting from continuous input into aquatic systems, combined with their potential for bioaccumulation, poses significant risks to aquatic ecosystems, and ultimately, to human health (Kang et al., 2022; Patel et al., 2019). Among these compounds, diclofenac (DCF), a widely used anti-inflammatory drug, has been frequently detected in wastewater at concerning concentrations, with literature reporting levels ranging from approximately 2 ng L^-1^–2,500 ng L^-1^ in effluent of Wastewater Treatment plants (WWTPs) (Dereszewska and Cytawa, 2023). Several studies have reported its presence in water bodies worldwide including Germany, United States, Japan, and India (Patel et al., 2019), highlighting the urgent need for effective removal solutions. Currently, conventional WWTPs have proven ineffective at removing pharmaceuticals, highlighting limitations in existing treatment processes (Silva et al., 2021).

In response, the European Commission published the revised Urban Wastewater Treatment Directive 2024/3019 (UWWTD) (EUR-Lex, 2024), which stipulates that by 2045, WWTPs serving populations greater than 150.000 population equivalents must implement a quaternary treatment, capable of removing at least 80% of the concentration at the inlet of a wide spectrum of microcontaminants, including DCF. Although the advanced quaternary treatments foreseen in the revised UWWTD aim to improved performance, the high cost of most available options hinders large-scale implementation. Consequently, the development of innovative, efficient, and cost-effective methodologies to reduce the presence of pharmaceuticals in wastewater is essential to meet future regulatory requirements.

Under these circumstances, adsorption based technology (Rocha et al., 2022; Silveira Neto et al., 2023) have emerged as a promising strategy. Adsorption processes are known for their easy implementation and high removal efficiency, while preventing the formation of potentially toxic by-products in the treated water, generating a saturated material, as microcontaminants are retained in a solid phase (Cheng et al., 2021). Different types of semi-continuous operation mode, such as stirred tanks (ST) (Rocha et al., 2022) and fixed-bed columns (FBC) (Jaria et al., 2019), can be used in adsorption-based wastewater treatments. The efficiency of these treatments largely depends on the performance of the adsorbent, making economic viability and the use of renewable resources key selection criteria. In this regard, biomass-derived carbon-based adsorbents, commonly referred to as biochar (BC), have gained attention due to their sustainability combined with high potential to remove microcontaminants from water (Correa-Navarro et al., 2020; Sousa et al., 2024).

From this perspective, the conversion of agricultural and industrial residues into BC represents a sustainable waste-valorisation strategy that enables the production of high-value-added materials. Spent brewery grains (SBG), the main by-product of the brewing process, account for approximately 85% of the solid waste generated during the production of beer, and require appropriate management solutions (Cancelliere et al., 2019; Carlini et al., 2021). Therefore, BC derived from SBG emerges as a sustainable alternative, enabling waste valorisation and its potential application in water treatment (Lynch et al., 2016).

Despite these advantages, BC presents limitations, namely the lack of selectivity towards specific target contaminants under competitive conditions (Arca-Ramos et al., 2017; Yu et al., 2024) and the need to deal with the exhausted material (Hassan et al., 2024). To overcome this challenge, BC modification strategies have been explored, particularly enzyme functionalization. In addition to improving selectivity, enzymes may also help mitigate or delay material saturation (Arca-Ramos et al., 2017). Among the enzymes explored for wastewater treatment, laccase (LAC, EC 1.10.3.2) has emerged as a promising biocatalyst (Arca-Ramos et al., 2017). In particular, LAC from *Trametes versicolor* is the most widely used, consistently exhibiting high degradation rates (often approaching 100% removal) for a broad range of pharmaceuticals, under optimized and controlled conditions (Debnath and Saha, 2020; Deska and Kończak, 2019). In the specific case of DCF, using free laccase (LAC_F_) at doses of 6000 U L^-1^ at an initial DCF concentration of 10 mg L^-1^ under room temperature (25 °C) and pH values between 4.5 and 6.9, highlighting the high efficiency and versatility of this enzyme (Adamian et al., 2021; Tran et al., 2010). However, the direct application of LAC_F_ in wastewater treatment is often limited by poor operational stability, difficulty in enzyme recovery, and lack of reusability. Immobilization of LAC onto solid supports provides a strategy to enhance enzyme stability and reusability (Wang et al., 2024; Zhou et al., 2021). The functionalised strategy aims to enhance DCF removal through a synergistic mechanism that combines the adsorption of the material toward DCF with enzymatic degradation promoted by LAC. In the present work, LAC was not intended to provide a highly selective binding mechanism toward DCF; instead, its immobilisation onto BC was expected to promote an enzymatic oxidation combined with an adsorption pathway.

In this context, this study evaluates DCF removal from buffered ultrapure water and urban wastewater by directly comparing the performance of a BC before and after functionalization with LAC (BC-LAC), under two semi-continuous adsorption systems. The use of semi-continuous configurations allows a more realistic assessment of process performance under conditions closer to those found in WWTP, overcoming key limitations associated with batch systems. While most previous works have focused on either adsorption or enzymatic degradation in only batch systems (Arca-Ramos et al., 2017; Correa-Navarro et al., 2020) and synthetic matrices (ultrapure water) (Correa-Navarro et al., 2020; Tran et al., 2010)This study integrates both approaches under dynamic operating conditions and in real wastewater. By doing so, it provides a more application-oriented evaluation of the effectiveness of enzyme-functionalized materials. To the best of our knowledge, this is the first work to investigate laccase-functionalized brewery-waste-derived BC in a semi-continuous adsorption system for pharmaceutical removal. Furthermore, this work offers new insights into the effectiveness of combined adsorption–biocatalytic approaches in dynamic systems.

## 2 Materials and methods

### 2.1 Reagents

The pharmaceutical used in the experiments was diclofenac sodium salt (DCF, ≥98%), supplied by TCI Europe. Laccase from *T. versicolor* (powder, LAC ≥0.5 U mg^-1^), diammonium salt of 2,2′-azino-bis (3-ethylbenzothiazoline-6-sulfonic acid) (ABTS ≥98%), anhydrous sodium acetate (CH_3_COONa ≥99%), and glacial acetic acid (CH_3_COOH, 100%) were obtained from Merck. Hydrochloric acid (HCl, 37%), purchased from Honeywell FLUKA, was used for material washing and pH adjustment. Sodium hydroxide solutions (NaOH, 99.3%) from José Manuel Gomes dos Santos were prepared for pH adjustment. For DCF quantification by High-Performance Liquid Chromatography with UV-Vis detection (HPLC-UV), acetonitrile (CH_3_CN, 99.9%) supplied by Fisher Scientific and phosphoric acid (H_3_PO_4_, 85.0%) from Acros Organics were used as the organic solvent and modifier, respectively.

All solutions used in this study were prepared using ultrapure water (18.2 MΩ cm^-1^, produced by a PURELAB Flex 4 system, ELGA VEOLIA) or secondary treated urban wastewater collected from the Aveiro Sul WWTP (Ílhavo, Portugal), as detailed in Section 2.2.

### 2.2 WWTP effluent sampling and characterization

The urban wastewater effluent used in this study was collected after secondary treatment at the Aveiro Sul urban WWTP, located in Ílhavo (Portugal), from the surface of the secondary clarifier. The collected samples correspond to the final effluent, i.e., the effluent to be discharged by the WWTP into receiving water bodies, which would constitute the target of an eventual advanced treatment stage if implemented. Two sampling campaigns were carried out in June and July 2025.

After collection, the effluent was further filtered through membrane filters with a pore size of 0.45 µm and a diameter of 293 mm (Gelman Sciences), in order to remove suspended particulate matter, improve matrix homogeneity, and reduce possible interference during the adsorption assays (Silva et al., 2025). During the collection process, sample temperature was not actively controlled due to the proximity between laboratory facilities and WWTP. The characterization of the effluent physicochemical parameters, as well as the corresponding results are described in Supplementary Material (SM), section SM1. The assessed parameters included pH, conductivity (µS cm^-1^), resistivity (MΩ cm^-1^), total dissolved solids (mg L^-1^), salinity (PSU), temperature (°C), and total organic carbon (TOC, mg L^-1^). The effluent was stored protected from light at 4 °C and used within a maximum period of 15 days (U.S. EPA, 2010).

### 2.3 Biochar production

Biochar (BC) derived from spent brewery grains (SBG) was produced following the procedure reported by Sousa et al. (2024). Briefly, SBG was air-dried at room temperature and milled using a manual blade grinder. Pyrolysis of the SBG was carried out in a microwave furnace (CEM Phoenix™ AirWave) at 800 °C for approximately 20 min, using a heating rate of 15 °C °min^-1^, under an inert N_2_ atmosphere (100 mL min^-1^). The resulting pyrolyzed SBG was washed with HCl (for ash removal), followed by washing with water until the pH of the recovered leachate was neutral. BC was then dried, in an oven, at 100 °C for 24 h. Before the operation in fixed-bed columns (FBC) and stirred tanks (ST), BC was sieved to obtain a granular material with particle size between 500–1000 µm.

The yield (η) of the produced material was calculated according to Equation 1:

$$\eta =\frac{Final\;BC\;mass}{Initial\;SBG\;mass}\times 100$$ (1)

where *Final BC mass* (g) is the mass of BC obtained after the entire washing and drying process, while *Initial SBG mass* (g) is the initial mass of SBG before thermal treatment.

### 2.4 Immobilization of laccase onto biochar

The immobilization of LAC onto the BC surface (yielding the BC-LAC material) was performed by physical adsorption, as described by (Almeida et al., 2026) Briefly, to this end, BC was suspended in a LAC solution with 5.0 mg mL^-1^, prepared in 0.1 M acetate buffer (acetic acid/sodium acetate) at pH 5.0. The suspension was magnetically stirred at 150 rpm and incubated at 30.0 °C for 24 h. After incubation, the composites were separated by filtration using paper filters (7–12 μm, Macherey-Nagel) and washed three times with 10 mL of 0.1 M acetate buffer at pH 5.0 (for unbound enzyme removal). The filtrate from each washing step was collected to determine residual enzymatic activity and assess the washing process’s efficiency. The resulting composite were recovered from the filters and dried at 40.0 °C for 48 h.

To minimize enzyme denaturation, LAC was immobilized onto BC by physical adsorption, under mild conditions. While the structural integrity of the immobilized enzyme was not directly assessed by spectroscopic techniques, the enzymatic activity measured through ABTS oxidation, indicates that at least part of the catalytic functionality of LAC was preserved after immobilization.

The LAC immobilization efficiency (%) was calculated from the difference between the initial LAC concentration in solution prior to incubation and the total LAC concentration quantified in all washing leachates. The LAC concentration in each leachate was determined, based on a calibration curve of absorbance at 420 nm per minute associated with ABTS oxidation, considering a LAC concentration range of 0.01–0.5 mg mL^-1^ (Almeida et al., 2026).

### 2.5 Materials characterization

The chemical and textural properties of the produced materials (BC and BC-LAC) were determined by N_2_ adsorption isotherms, for the determination of the specific surface area ( $S_{BET}$ ), and scanning electron microscopy (SEM). The point of zero charge (PZC) was determined only for BC, whereas enzymatic activity was assessed only for BC-LAC. The experimental procedures used for characterization of the materials are described in Supplementary Material, section SM2.

### 2.6 Batch adsorption experiments–kinetic and equilibrium studies

Batch adsorption experiments were performed for BC with DCF to determine adsorption kinetics and equilibrium parameters, which are essential for the design and optimization of the subsequent semi-continuous adsorption systems. DCF solutions (5 mg L^-1^), prepared in 1 mM acetate buffer at pH 7.0 were brought into contact with BC in polypropylene tubes. For each assay, 10 mL of DCF solution was contacted with accurately weighed amounts of BC, with the adsorbent dose selected according to the type of experiment, as described below. The suspensions were shaken on an overhead shaker (Reax 2, Heidolph), at 80 rpm under controlled temperature (25.0 °C ± 0.1 °C). Control experiments containing DCF solutions without adsorbent were conducted in parallel. To enable monitoring of DCF concentration in the aqueous phase during the experiments with WWTP effluent and considering the limit of detection of the analytical methodology employed (described in SM3), the previously filtered effluent was spiked with DCF to establish an initial concentration of 5 mg L^-1^. Spiking was performed immediately before the beginning of each experiment, ensuring solution homogeneity by ultrasonic treatment (Ultrasounds HD, P-Selecta) for 1 h. After the contact time, the samples were filtered through 0.22 µm syringe filters (PVDF, Whatman), to remove fine suspended particles of adsorbent and matrix solids, thus minimizing interference during HPLC-UV analysis (Aldeguer Esquerdo et al., 2021), and the residual DCF concentration in solution was determined by HPLC-UV, as described in SM3.

Kinetic adsorption studies were conducted to determine the time needed to reach equilibrium. These experiments were performed using a fixed BC dose of 300 mg L^-1^ (3.0 mg per assay), while maintaining a constant solution volume (10 mL) and the initial DCF concentration (5 mg L^-1^). Adsorption was monitored over contact times ranging from 2 to 72 h. The experimental kinetic data were fitted to pseudo-first-order (Ferreira, 2017), pseudo-second-order (Ferreira, 2017), Elovich (Nascimento et al., 2014) and intraparticle diffusion (Qiu et al., 2009) models, whose governing equations are provided in Supplementary Material SM4.

Equilibrium adsorption experiments were subsequently carried out using different BC doses, ranging from 250 to 1150 mg L^-1^, while maintaining a constant solution volume (10 mL) and initial DCF concentration (5 mg L^-1^). A contact time of 24 h was adopted to ensure equilibrium conditions. After determining the equilibrium time, equilibrium adsorption experiments were carried out to evaluate the adsorption performance of BC by varying the adsorbent dose between 250 and 1150 mg L^-1^, while keeping constant the DCF concentration (5 mg L^-1^), solution pH (7.0) and contact time of 24 h. The experimental data were fitted to Langmuir (Nascimento et al., 2014), Freundlich (Nascimento et al., 2014), Langmuir-Freundlich (Ferreira, 2017) and Linear-Langmuir (Murphy et al., 2023) isotherm models, with the corresponding equations detailed in Supplementary Material SM4. Non-linear regression analysis of both kinetic and equilibrium data was performed using GraphPad Prism Software (Version 8.0.1).

### 2.7 Semi-continuous adsorption studies

#### 2.7.1 Fixed-bed column experiments

**FIGURE 1:**
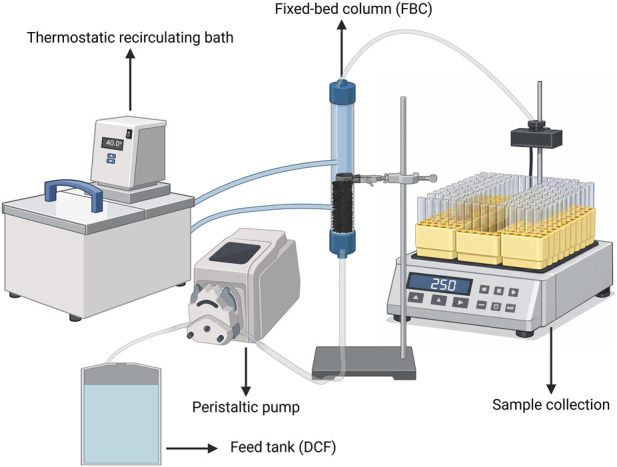
Schematic representation of the fixed-bed column (FBC) experimental setup, including feed tank with DCF, peristaltic pump, CHROMAFLEX® glass column packed with BC, thermostatic recirculating bath, and sample collection unit. (Created in BioRender. Gouveia, R. (2026) https://BioRender.com/elhitbk).

All FBC experiments were carried out in a Kimble® Chromaflex® glass column with a height of 15 cm and an internal diameter of 2.5 cm, equipped with an acrylic jacket and maintained at a constant temperature of 25 °C ± 1 °C using a recirculating thermostatic water bath (Haake A10, Thermo Scientific), as shown in Figure 1 and Supplementary Figure S1 of the SM. The column was packed with different amounts of BC, resulting in different bed heights, which were subsequently adjusted using a flow adapter and a high-density polyethylene (HDPE) bed support (20 µm porosity) at the top of the column. Prior to each experiment, the system was equilibrated for approximately 24 h with a continuous flow of ultrapure water to remove air trapped in the fixed-bed (Jaria et al., 2019).

The DCF solution was pumped upward through the FBC using a peristaltic pump (BT100-1L/YZ II 15, 2 channels, Longer Pump). The column effluent was collected at predefined time intervals until the DCF concentration reached a constant value, using a programmable fraction collector (IS-95 Interval Sampler, Spectra/Chrom®). The concentration of DCF in the aqueous phase was determined by HPLC-UV according to the procedure described in SM3.

Unlike batch assays, in which a fixed volume is contacted with the adsorbent in a closed system, the removal of DCF by adsorption onto BC in the FBC, operated in semi-continuous mode, and was investigated in three successive stages, with performance evaluated through breakthrough curves. Although the column was operated under continuous flow during each experiment, the overall operation can be described as semi-continuous, since the system was periodically stopped between experimental stages to modify operating conditions. First, the effect of adsorbent mass (1.0, 1.5, and 2.0 g), corresponding to different bed heights, was assessed using ultrapure water buffered at pH 7.0. Subsequently, using the most favourable adsorbent mass, the influence of the flow rate (6.94 ⋅ $10^{-4}$ , 1.39 ⋅ $10^{-3}$ and 2.78 ⋅ $10^{-3}$ L min^-1^) was studied under the same conditions. Finally, the performance of BC and BC-LAC was evaluated using urban WWTP effluent, under optimized operating conditions selected from the previous studies (1.0 g L^-1^ and 1.0 L d^-1^). For BC-LAC, the column feed was additionally subjected to a continuous flow of compressed air at a pressure of 2 bar, as illustrated in Supplementary Figure S2 of the SM.

#### 2.7.2 Stirred tank experiments

**FIGURE 2:**
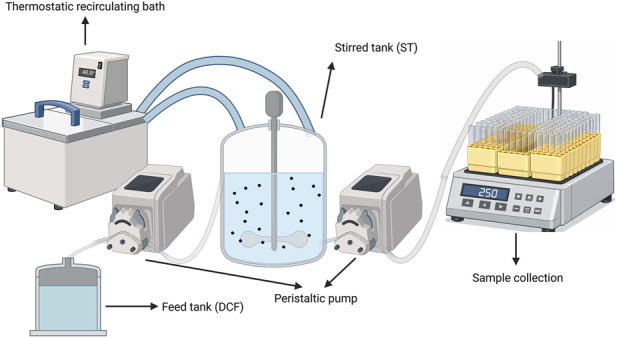
Schematic representation of the stirred tank (ST) experimental setup, including feed tank, peristaltic pump, tank with BC, thermostatic recirculating bath, and sample collection unit. (Created in BioRender. Gouveia, R. (2026) https://BioRender.com/qt2q7kd).

A cylindrical tank with an internal diameter of 114 mm and a height of 160 mm was used for these experiments. The ST was surrounded by an acrylic jacket with an external diameter of 150 mm and a height of 185 mm for temperature control at 25 °C ± 1 °C, using a thermostat water recirculation bath (HAAKE A10, Thermo Scientific). The tank was fed from the top with DCF solution, and the treated solution was collected from the bottom. To ensure equal inlet and outlet flow rates, two peristaltic pumps (BT100-1L/YZ II 15, 2 channels, Longer Pump) were used. The experimental configuration is illustrated in Figure 2 and in the SM, Supplementary Figure S3.

Adsorption experiments in the ST were conducted after filling the tank with 1.2 L of the selected aqueous matrix and a fixed dose of the material. Mixing was ensured by a vertical shaft stirrer (Hei-TORQUE Value 100, Heidolph) equipped with a steel impeller (BR10 cross blade impeller, Heidolph), operated continuously at 200 rpm to guarantee a complete mix of the suspended material. The ST design, along with the impeller position and rotational speed, was carefully selected to achieve homogeneous dispersion while preventing particle sedimentation. During the tests, before sample collection, the samples were passed through a 0.45 µm membrane filter (SUPOR®, Gelman Sciences), followed by a stainless-steel mesh installed downstream of the filter and fixed at the bottom of the ST. After, the samples were collected at predefined time intervals until the DCF concentration at the outlet remained constant, using a programmable sample collector (IS-95 Interval Sampler, Spectra/Chrom®). The collected samples were analysed to determine the DCF concentration, as described in section SM3 in SM.

Like the FBC, adsorption studies of DCF onto BC in ST operating mode were conducted in three successive stages. Initially, to evaluate the effect of BC dose (0.5, 1.0, and 1.5 g L^-1^), the tank was fed with a DCF solution at 5.0 mg L^-1^ prepared in ultrapure buffered water (pH 7.0), at a flow rate of 1.39 ⋅ $10^{-3}$ L min^-1^. Subsequently, flow rates of 6.94 ⋅ $10^{-4}$ , 1.39 ⋅ $10^{-3}$ and 2.78 ⋅ $10^{-3}$ L min^-1^ were tested, feeding the tank with DCF solution (5.0 mg L^-1^) and using a BC dose of 0.5 g L^-1^. Finally, using the optimised operating conditions (0.5 g L^-1^ and 6.94 ⋅ $10^{-4}$ L min^-1^), the performance of BC and BC-LAC was evaluated using WWTP effluent. In the case of BC-LAC, the feed was continuously aerated with compressed air at 2 bars. A schematic representation is provided in Supplementary Figure S4 of SM.

#### 2.7.3 Data analysis and modelling

In FBC systems, the breakthrough curve represents the ratio between the solute concentration at the system outlet (*C*) and the influent concentration (*C*_0_) as a function of time. This curve indicates the point at which the adsorbent begins to lose efficiency, as well as the time required to reach saturation (Ferreira, 2017). In ST systems, which are ideally perfectly mixed, adsorption performance is instead described by the dynamic evolution of C over time, reflecting the transient response of the system, known as time-course of concentration (Fogler, 2013). According to EU Directive 2024/3019 (EUR-Lex, 2024), a ratio of 20% (*C*/*C*_0_ = 0.2) was used to define the breakthrough point. For this purpose, the breakthrough time (*t*_*b*_, i.e., the time at which the ratio between C and $C_{0}$ equals 0.2), the saturation time (*t*_*s*_, i.e., the time at which *C*/*C*_0_ reaches a constant value), and the treated volume at breakthrough (*V*_*b*_) were determined.

Semi-empirical kinetic models based on mass balance equations are used to describe and fit breakthrough curves, enabling the extraction of key parameters for adsorption system analysis and design (de Franco et al., 2017; Jaria et al., 2019).

The model proposed by Yan et al. (2001) offers greater flexibility for systems experiencing significant variations in operating conditions, such as very short or very long operating times. This model has been widely used in environmental studies to describe the adsorption of contaminants onto various adsorbents (de Franco et al., 2017). Equation 2 represents the Yan et al. model:

$$\frac{C}{C_{0}}=\frac{1}{1+{\left(\frac{C_{0}\cdot Q\cdot t}{q_{\gamma }\cdot m}\right)}^{\alpha_{\gamma }}}$$ (2)

where Q (L min^-1^) is flow rate, t (min) is the time, $q_{\gamma }$ (mg g^-1^) is the amount of solute adsorbed, m (g) is the mass of adsorbent and $\alpha^{\gamma }$ is a model parameter. This model was used for analysing the breakthrough curves obtained from FBC operation and the dynamic behaviour of the ST. For simplicity, the term breakthrough curves will be considered to refer to both cases, in further discussion.

Other models were also evaluated, like Thomas model (de Franco et al., 2017) and Yoon-Nelson model (Ferreira, 2017) (following the approach proposed by Ferrero et al., as reported in the study of Rocha et al., (2022), however they did not provide a satisfactory fit to the experimental data.

## 3 Results and discussion

### 3.1 Biochar production

The calculated yield for BC production was 26% before sieving and 15% after sieving for the particle size fraction of interest in this work (500–1000 µm). Particle-size selection was addressed, given its critical role in adsorbent performance and system behaviour (Debnath and Saha, 2020; Rocha et al., 2022). Powered particles (diameter <180 µm) are suitable for ST, in which particle suspension is maintained. Under these conditions, the high surface promotes higher adsorption rates, allowing equilibrium to be reached in a short operation time, while minimizing risks of sedimentation or systems clogging (Rocha et al., 2022). In contrast, in FBC, the use of particles with such fine granulometry may result in high pressure drop, particle deposition, and bed clogging, thereby compromising system performance. In this configuration, the use of granular adsorbents with particle sizes larger than 180 µm is recommended to ensure bed stability, efficient fluid flow, and easier regeneration (Ferreira, 2017; Rocha et al., 2022). Accordingly, a range of adsorbent particle sizes between 500 and 1000 µm was selected to balance efficiency, stability, and versatility in both systems. The same particle-size was selected for both systems to facilitate a more direct comparison of the results. In the study by Sousa et al. (2020), BC produced from brewery spent grain via microwave-assisted pyrolysis exhibited yields of approximately 30%, considering all particle size fractions. These values are close to those obtained in the present work and therefore represent a relevant contribution to future research.

### 3.2 Immobilization of laccase onto biochar

Understanding the immobilization mechanism requires a combined analysis of the PZC of BC (3.9) and the isoelectric point of purified LAC from *T. versicolor* (around 5.8 (Bertrand et al., 2014)). The immobilization efficiency achieved was 6.1%. Although at pH 5.0, electrostatic interactions between the enzyme and the BC surface may be expected, however, variation in reported laccase isoelectric points depending on their origin and preparation conditions should be considered. Therefore, electrostatic alone cannot fully explain the limited immobilization efficiency observed. This suggests that, in addition to electrostatic forces, other factors influence enzyme fixation, such as the available surface area of BC, the suitability of the LAC loading strategy, and support saturation. Additionally, hydrophobic interactions, hydrogen bonding, or π-π interactions between the aromatic functional groups of the enzyme and the BC surface may also play a relevant role.

### 3.3 Materials characterization

N_2_ adsorption/desorption isotherms were used to determine the textural parameters of the produced materials (BC and BC-LAC). The $S_{BET}$ was calculated by the BET method, while micropore parameters were obtained using the Dubinin-Astakhov model, as described in Section 2.5. It is important to highlight that the BET method is based on gas-phase adsorption and was therefore used exclusively for textural characterization, whereas the adsorption of DCF in aqueous solution was described using liquid-phase equilibrium models. The results are summarized in Table 1.

**TABLE 1:** Textural parameters of the produced materials (BC and BC-LAC): specific surface area ( $S_{BET}$ ), total pore volume ( $V_{p}$ ), micropore volume ( $W_{0}$ ), average micropore width (L), and average pore diameter ( $D_{p}$ ).

Nitrogen adsorption at −196 °C					
Sample	$S_{BET}$ (m^2^ g^-1^)	$V_{p}$ (cm^3^ g^-1^)	$D_{p}$ (nm)	Dubinin-Astakhov	
				$W_{0}$ (cm^3^ g^-1^)	L (nm)
BC	301	0.14	0.95	0.12	1.48
BC-LAC	191	0.10	1.06	0.07	1.66

The BC exhibited a relatively high surface area, particularly when compared with non-activated BC reported in recent literature (Irewale et al., 2025; Wystalska and Kwarciak-Kozłowska, 2021), considering the absence of any activation procedure ( $S_{\text{BET}}=301$ m^2^ g^-1^) and a moderate total pore volume ( $V_{p}$ = 0.14 m^3^ g^-1^), as well as an average pore diameter ( $D_{p}$ = 0.95 nm) compatible with a well-developed porous structure. This result can be attributed to the microwave-assisted pyrolysis conditions, which promote differences in heat and mass transfer, favouring efficient volatilization of the material and, consequently, an increase in porosity compared to a conventional pyrolysis system.

Accordingly, Sousa et al. (2020) reported an $S_{\text{BET}}$ of 3 m^2^ g^-1^ for BC produced from SBG by conventional pyrolysis, which reinforces the higher efficiency of microwave-assisted pyrolysis in obtaining materials with well-developed textural properties.

After LAC immobilization, the ${\;S}_{BET}$ decreased to 191 m^2^ g^-1^, accompanied by a reduction in pore volume ( $V_{p}$ = 0.10 m^3^ g^-1^). These values, lower than those of non-functionalized BC, indicate that LAC likely covered and blocked a significant portion of the surface and porous structure, resulting in pore obstruction and limiting access to the internal structure (Adamian et al., 2021). Overall, the results confirm the efficiency of microwave-assisted pyrolysis in generating BC with moderately high developed porosity from SBG, despite the absence of a chemical activation process. However, the immobilization by physical adsorption led to a significant decrease in $S_{BET}$ , likely due to the blockage of meso and macropores by the enzyme, thereby reducing the characteristic porosity of the material.

**FIGURE 3:**
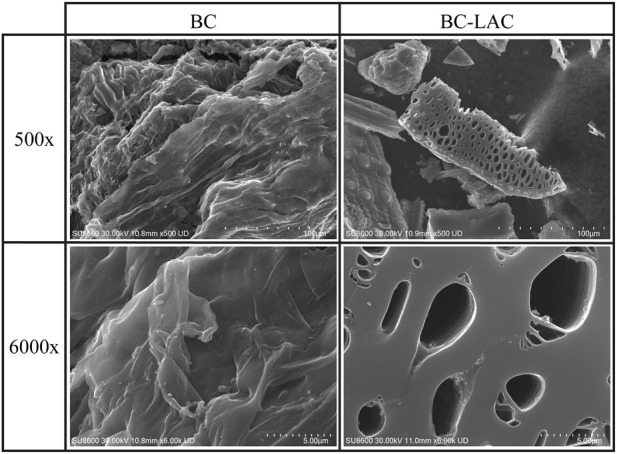
SEM images of BC and BC-LAC at magnifications of 500x and 6000x.

Figure 3 presents SEM images of BC and BC-LAC at magnifications of 500x and 6000x. Figure 3 shows SEM images of BC and BC-LAC displaying a fragmented and irregular morphology with rough surfaces and a layered organization, characteristic of thermal degradation during microwave-assisted pyrolysis. In the case of BC-LAC, numerous deep, rounded cavities with well-defined edges are clearly observed, giving rise to a more open surface appearance. These observations indicate that enzymatic immobilization induces noticeable morphological modifications compared to the more compact and lamellar structure of the unmodified BC. Given the low enzyme loading, the presence of these cavities suggests that the changes are not solely attributable to the immobilized laccase, but also to the immobilization procedure itself. Contact with buffer solutions, agitation, and successive washing steps may have promoted surface restructuring or the exposure of previously inaccessible surface features.

Considering the molecular size of laccase, partial blockage of micro- and mesopores during immobilization is plausible and may account for the observed decrease in *S*_BET_, even if larger surface cavities become more apparent at the higher scale of SEM.

The PZC of the unmodified BC was determined using the pH drift method, yielding a value of 3.9. This result indicates that at pH values above this threshold, BC exhibits a predominantly negative surface charge, associated with the deprotonation of acidic surface functional groups. The PZC was determined only for non-functionalized BC, as the application of this technique to enzyme-modified materials would not yield a representative or easily interpretable value. The pH drift method assumes a solid surface with functional groups that ionize in a predictable and static manner as a function of pH. However, LAC is a protein containing multiple ionizable groups (e.g., carboxyl and amine), whose protonation state depends on the molecule, which exhibits complex ionization behaviour dependent on the molecule’s conformation and interactions with the surrounding medium. This continuous structural reorganization hinders the definition of a stable and meaningful PZC for the composite material. In addition, the method requires exposure to a wide pH range, which may induce enzyme denaturation and compromise its three-dimensional structure and catalytic activity (Robinson, 2015).

This behaviour may be associated with enzymatic self-shielding phenomena, in which immobilized enzyme molecules limit substrate access to active sites, as well as molecular overlap or steric hindrance, where molecular size physically restricts substrate interaction (Khan, 2021). These results suggest that physical adsorption alone may not be enough to achieve high levels of effective enzyme functionalization on BC.

### 3.4 Batch adsorption experiments–kinetic and equilibrium studies

**FIGURE 4:**
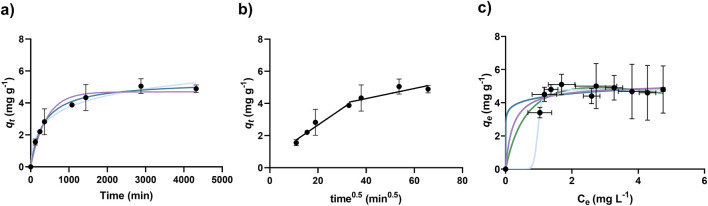
**(a)** Kinetic studies of DCF adsorption onto BC in acetate buffer at pH 7.0. The experimental data were fitted to the pseudo-first order (purple), pseudo-second order (blue), Elovich (light blue) and **(b)** intraparticle diffusion models. **(c)** Equilibrium studies of DCF adsorption onto BC in acetate buffer at pH 7.0. The experimental data were fitted to the Langmuir (purple), Freundlich (blue), Langmuir–Freundlich (light blue), and Linear-Langmuir (emerald) isotherm models. Error bars represent the standard deviation (n = 3). Experimental conditions: initial DCF concentration ([DCF]ᵢ) = 5 mg L^-1^ | adsorbent dose = 300 mg L^-1^ (kinetic assays) | contact time = 3600 min (equilibrium assays) | 25.0°C ± 0.1°C | 80 rpm.

Figure 4a,b show the experimental kinetics data for DCF removal by BC and their fitting to the applied kinetic models. Figure 4c shows the adsorption isotherms, relating the amount of DCF adsorbed at equilibrium (*q*_*e*_, mg g^-1^) to its residual concentration in solution (*C*_*e*_, mg L^-1^). Fitting parameters of the kinetic and equilibrium models to the experimental results are present in Table 2.

**TABLE 2:** Fitting parameters of kinetic and equilibrium models for the experimental data of DCF adsorption onto BC under the experimental conditions: [DCF]ᵢ = 5 mg L^-1^ | Dose = 300 mg L^-1^ (kinetics) |Time = 3600 min (equilibrium) | 25.0 °C ± 0.1 °C | 80 rpm.

Model	Fitting paraments	Values
Kinetic experiments		
Pseudo-first order	$q_{e}$ (mg g^-1^)	4.7 ± 0.2
	$k_{1}$ (min^-1^)	(2.5 ± 0.4)·10^−3^
	*R* ^2^	0.973
	*S*_y/x_	0.316
Pseudo-second order	$q_{e}$ (mg g^-1^)	5.4 ± 0.1
	$k_{2}$ (min^-1^)	(5.6 ± 0.6)·10^−4^
	*R* ^2^	0.994
	*S*_y/x_	0.152
Elovich	α	0.035 ± 0.008
	β	0.9 ± 0.7
	*R* ^2^	0.987
	*S*_y/x_	0.217
Intraparticular diffusion	$k_{id\left(1\right)}$ (mg g ^-1^ min^0.5^)	0.10 ± 0.01
	$k_{id\left(2\right)}$ (mg g ^-1^ min^0.5^)	0.03 ± 0.02
	*R* ^2^_(1)_	0.961
	*R* ^2^_(2)_	0.768
	*S*_y/x(1)_	0.238
	*S*_y/x(2)_	0.318
Equilibrium experiments		
Langmuir	$q_{\max}$ (mg g^-1^)	5.1 ± 0.3
	$K_{L}$ (L mg^-1^)	4.5 ± 3
	*R* ^2^	0.931
	*S*_y/x_	0.405
Freundlich	$K_{F}$ (mg g^-1^ (L mg^-1^)^−n^)	4.3 ± 0.3
	n	12 ± 8
	*R* ^2^	0.923
	*S*_y/x_	0.429
Langmuir-freundlich	$q_{\max}$ (mg g^-1^)	4.8 ± 0.8
	$K_{LK}$ (L mg^-1^)^−n^)	1.5 ± 0.5
	n	0.07 ± 0.03
	*R* ^2^	0.983
	*S*_y/x_	0.211
Linear-Langmuir	*m*	−0.5 ± 0.4
	$q_{\max}$ (mg g^-1^)	8 ± 9
	$K_{LL}$ (L mg^-1^)	1.3 ± 0.9
	*R* ^2^	0.946
	*S*_y/x_	0.381

As shown in Figure 4a, the adsorption rate of DCF in BC was relatively slow, with equilibrium reached only after 1440 min, which may be attributed to diffusional limitations in the transport of DCF to active sites of BC. Additionally, electrostatic repulsion between the anionic DCF (pka ≈4.15 (Diclofenac, 2026)) and the negatively charged BC surface at pH 7.0 (PZC = 3.9) reduces the overall affinity between adsorbate and absorbent, thereby limiting both adsorption capacity and rate. Particle size and pore distribution of BC may further hinder diffusion within the porous structure.

As shown in Table 2, the experimental data were fitted to several kinetic models, with the pseudo-second-order model providing the best fit (*R* ^2^ = 0.994; *S*_y/x_ = 0.152). Although often associated with chemisorption, this fitting more likely reflects surface-controlled kinetics, given the unfavourable electrostatic interactions between DCF and BC.

Regarding the equilibrium experiments, the Langmuir-Freundlich model provided the best fit to the equilibrium data (*R* ^2^ = 0.983; *S*_y/x_ = 0.211), suggesting predominantly monolayer adsorption on energetically heterogeneous surfaces, with a *q*_m_ of 4.8 ± 0.8 mg g^-1^. In contrast, the lower goodness-of-fit obtained for Langmuir and Freundlich models reflects the non-ideal nature of adsorption systems. However, the relatively high uncertainty associated with the estimated parameters, the limited number of experimental points at low concentrations, and reported thermodynamic inconsistencies of this model in this concentration range (Buttersack, 2019) should be considered. Nevertheless, the isotherm profile exhibits a clear plateau (*cf.* Figure 4c), indicating saturation of adsorption sites.

Overall, BC demonstrated relevant adsorption performance, achieving 79.3% DCF removal at a dose of 1150 mg L^-1^. Although adsorption efficiency is constrained by unfavourable electrostatic interactions at the studied pH, these results confirm the suitability of BC as an effective adsorbent.

### 3.5 Semi-continuous adsorption studies

#### 3.5.1 Fixed-bed column experiments

##### 3.5.1.1 Effect of adsorbent mass

**FIGURE 5:**
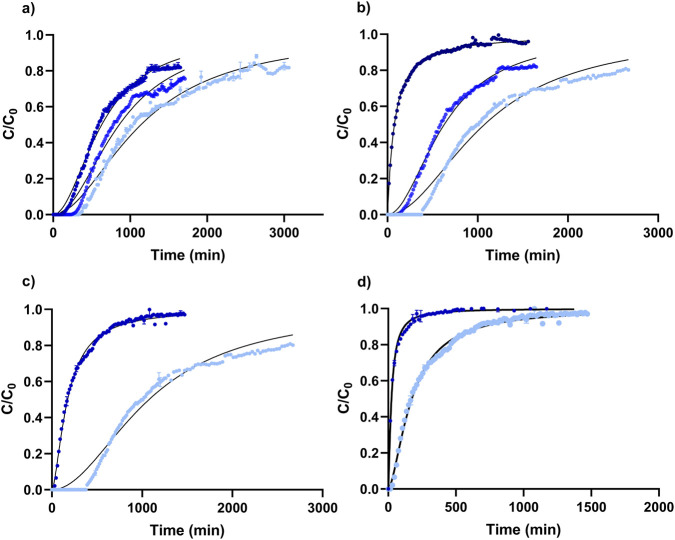
Breakthrough curves of the experimental data for DCF adsorption in the FBC: **(a)** effect of different BC masses, namely 1.0 g (dark blue), 1.5 g (medium blue), and 2.0 g (light blue); **(b)** effect of different flow rates, specifically 6.94 ⋅ $10^{-4}$ L min^-1^ (light blue), 1.39 ⋅ $10^{-3}$ L min^-1^ (medium blue), and 2.78 ⋅ $10^{-3}$ L min^-1^ (dark blue); **(c)** DCF adsorption onto BC using buffered water at pH 7.0 (light blue) and real effluent (dark blue); **(d)** DCF adsorption onto BC (light blue) and BC-LAC (dark blue) in real effluent. The experimental data were fitted to the Yan et al. model (black line). Error bars represent the standard deviation (n = 3).

The adsorption of DCF onto BC in FBC was investigated under various operating conditions to evaluate the influence of adsorbent mass. The breakthrough curves presented in Figure 5a, obtained under dynamic conditions, show that DCF adsorption increased with increasing BC mass in the column. A minimum DCF removal efficiency of 80% relative to the influent was adopted, and the corresponding values are reported in Table 3. The Yan et al. model was fitted to the experimental data, with the corresponding parameters summarized in Table 4.

**TABLE 3:** Characteristic parameters of FBC adsorption at different BC masses (1.0, 1.5, and 2.0 g), different influent flow rates (6.94 ⋅ $10^{-4}$ , 1.39 ⋅ $10^{-3}$ and 2.78 ⋅ $10^{-3}$ L min^-1^) and for different matrix (buffered water at pH 7.0 and wastewater) including stoichiometric time (t_s_), breakthrough time (t_b_), treated volume (V_b_), and length of unused bed (LUB).

Parameters	BC mass (g)^a^			Flow rate (L min^-1^)^b^			Matrix^c^	
	1.0	1.5	2.0	6.94 ⋅ $10^{-4}$	1.39 ⋅ $10^{-3}$	2.78 ⋅ $10^{-3}$	Buffered water pH 7.0	Wastewater
$t_{s}$ (min)	758	950	1198	1308	758	199	1307.8	273.8
$t_{b}$ (min)	326	465	611	596	326	19	596.8	72.7
$V_{b}$ (L)	0.45	0.67	0.83	0.42	0.46	0.08	0.42	0.25
LUB (cm)	1.4	1.9	3.0	1.7	1.4	4.3	1.7	3.1

a Experiments conducted at a constant flow rate of 1.394 ⋅ $10^{-3}$ L min-1.

b Experiments conducted at constant adsorbent dose of 1.0 g.

c Experiments conducted at a constant flow rate of 6.94 ⋅ $10^{-4}$ L min-1 and constant adsorbent dose of 1.0 g.

**TABLE 4:** Parameters obtained from fitting the Yan et al. model to the experimentally obtained breakthrough curves, in buffered water at pH 7.0, for different BC masses (1.0, 1.5, and 2.0 g) in the FBC system and for different BC doses (0.5, 1.0, and 1.5 g L^-1^) in the ST system, with a fixed flow rate of 1.394 ⋅ $10^{-3}$ L min^-1^.

Model	Parameters	FBC		ST		
		BC mass (g)			BC-LAC doses (g L^-1^)		
		1.0	1.5	2.0	0.5	1.0	1.5
Yan et al.	*α*_*Y*_	1.96 ± 0.03	2.1 ± 0.1	1.92 ± 0.04	1.83 ± 0.03	1.86 ± 0.02	2.05 ± 0.04
	*q*_*Y*_ (mg g^-1^)	3.67 ± 0.03	4.99 ± 0.05	6.64 ± 0.06	4.62 ± 0.03	3.15 ± 0.02	2.42 ± 0.02
	*R* ^2^	0.991	0.974	0.980	0.991	0.996	0.988
	*S*_y/x_	0.027	0.045	0.042	0.027	0.020	0.036

The results indicate longer operating times and larger treated water volumes for 2.0 g of BC, followed by 1.5 g and 1.0 g. These findings can be attributed to the higher number of available sites when larger adsorbent masses are used, delaying column saturation. The parameters $q_{\gamma }$ and $a_{\gamma }$ obtained from the model provided a reliable description of column performance. Although the highest BC mass (2.0 g) resulted in the greatest adsorption capacity ( $q_{\gamma }$ = 6.64 ± 0.06 mg g^-1^), followed by 1.5 g ( $q_{\gamma }$ = 4.99 ± 0.05 mg g^-1^) and 1.0 g ( $q_{\gamma }$ = 3.67 ± 0.03 mg g^-1^), the final selection was 1.0 g of BC. This decision was supported by the fact that, although higher adsorbent masses extended the operating time and resulted in higher treated volumes, diminishing performance gains relative to the additional material required were observed. Therefore, a BC mass of 1.0 g was considered the most appropriate.

##### 3.5.1.2 Effect of flow rate

The effect of flow rate on FBC performance was also evaluated, and the breakthrough curves shown in Figure 5b indicates that DCF adsorption onto BC increases as the flow rate decreases, showing a good fit to Yan et al. model.

The obtained breakthrough curves exhibit a steeper slope during the initial adsorption stage, which is characteristic of this type of system due to the greater availability of active sites at the beginning of the process. Small variations in BC particle size may lead to differences in packing density, resulting in preferential flow paths and variations in mass transfer. In addition, the shape of the breakthrough curve is influenced by the adsorption rate and by mass transfer from the liquid phase to the active sites within the BC particles.

The parameters derived from the breakthrough curve are presented in Table 3. A flow rate of 6.94 ⋅ $10^{-4}$ L min^-1^ resulted in the longest operating time, whereas the higher *V*_*b*_ was obtained at 1.39 ⋅ $10^{-3}$ L min^-1^, corresponding to 0.42 L in 546 min compared with 0.46 L in 326 min. Although lower, the *V*_*b*_ achieved at 6.94 ⋅ $10^{-4}$ L min^-1^ was very close to that obtained at 1.39 ⋅ $10^{-3}$ L min^-1^, while being achieved more gradually. In contrast, at 2.78 ⋅ $10^{-3}$ L min^-1^, breakthrough occurred quickly, reaching complete bed exhaustion (C/C_0_ = 1), indicating total column saturation. These results show that a flow rate of 6.94 ⋅ $10^{-4}$ L min^-1^ significantly extends the *t*_*b*_ and ensures more efficient bed utilization, whereas 1.39 ⋅ $10^{-3}$ L min^-1^ allows a slightly *V*_*b*_. However, in complex matrices such as wastewater, a flow rate of 1.39 ⋅ $10^{-3}$ L min^-1^ might tend to accelerate column saturation. Thus, considering all results and the previous analysis, a flow rate of 6.94 ⋅ $10^{-4}$ L min^-1^ was selected as the most suitable condition for subsequent experiments, ensuring longer operating time and more efficient bed utilization, under realistic conditions.

##### 3.5.1.3 Experiments with wastewater and immobilized laccase

The impact of feed matrix on the performance of BC for DCF adsorption in FBC was subsequently evaluated by comparing buffered ultrapure water at pH 7.0 with real wastewater effluent. Figure 5c highlights the strong effect of the matrix on FBC performance. Comparison between the two matrices shows that the column fed with buffered ultrapure water at pH 7.0 exhibited a higher adsorption capacity, whereas DCF was detected earlier at the column outlet when effluent was used, confirming accelerated saturation despite identical operating conditions.

As shown in Table 3, *t*_*b*_ and, consequently, column saturation occurred much faster in effluent than in buffered water at pH 7.0 (72.7 min vs. 596.8 min). Both the *t*_*b*_ and the treated water volume were approximately eight times lower, and a higher length of unused bed was observed, reflecting less efficient column utilization. This behaviour can be mainly attributed to the higher complexity of the effluent, which promotes competition for active sites between DCF, and other solutes present in the matrix, such as dissolved organic matter (15.87 ± 0.08 ppm), metal cations, and other organic contaminants. These constituents may also contribute to partial pore blockage of BC, thereby reducing the overall adsorption efficiency in the FBC. Variations in effluent pH may additionally influence surface charge and intensify electrostatic repulsion, however this effect is considered secondary compared to presence of multiple competing constituents.

The performance of BC-LAC in effluent under FBC operation is shown in Figure 5d, and as observed in previous assays, the Yan et al. model provided the best fit to the experimental data. Under identical operating conditions but using BC alone, a higher *t*_*b*_ was observed, reflecting a longer operating time, a larger *V*_*b*_, and a reduced length of unused bed, when compared with BC-LAC system. The column packed with BC-LAC reached breakthrough in an operation time lower than 15 min, treating a substantially lower effluent volume than BC. It should be noted that a direct comparation of *V*_*b*_ at *C/C_0_* = 0.2 was not possible, since BC-LAC data are only available from the end of the first sampling interval (15 min), corresponding to a *C/C_0_* = 0.38. Nevertheless, considering this operating point, the volume treated with BC was approximately five times higher than that obtained with BC-LAC, indicating that laccase immobilization did not improve column performance and instead resulted in a marked reduction in DCF removal efficiency. This behaviour can be explained by a combination of structural and functional factors. Enzyme immobilization likely caused partial blockage of micro- and mesopores, which are primarily responsible for adsorption capacity, as evidenced by the decrease in S_BET_. Consequently, access of DCF molecules to the internal porous network was restricted, reducing the effective number of adsorption sites. Additionally, the contribution of enzymatic degradation under fixed-bed conditions was likely limited. Laccase is an oxidoreductase that requires molecular oxygen as a co-substrate, and although compressed air was continuously injected into the feed, the absence of additional oxygen inputs along the column may have constrained oxygen transport and diffusion within the packed bed. This limitation could have hindered enzymatic activity, thereby reducing the potential synergistic effect between adsorption and biocatalysis during column operation.

#### 3.5.2 Stirred tank experiments

##### 3.5.2.1 Effect of adsorbent dose

**FIGURE 6:**
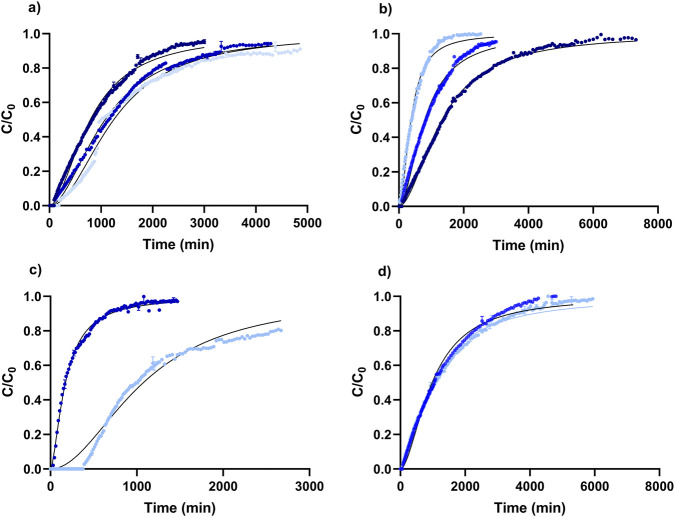
Breakthrough curves of the experimental data for DCF adsorption in the ST: **(a)** effect of different BC doses, namely 0.5 g L^-1^ (dark blue), 1.0 g L^-1^ (medium blue), and 1.5 g L^-1^ (light blue); **(b)** effect of different flow rates, specifically 6.94 ⋅ $10^{-4}$ L min^-1^ (dark blue), 1.39 ⋅ $10^{-3}$ L min^-1^ (medium blue), and 2.78 ⋅ $10^{-3}$ L min^-1^ (light blue); **(c)** DCF adsorption onto BC using buffered water at pH 7.0 (light blue) and real effluent (dark blue); **(d)** DCF adsorption onto BC (light blue) and BC-LAC (dark blue) in real effluent The experimental data were fitted to the Yan et al. model (black line). Error bars represent the standard deviation (n = 3).

The effect of BC dose on DCF adsorption in the ST was evaluated for doses of 0.5, 1.0 and 1.5 g L^-1^, using a feed flow rate of 1.39 ⋅ $10^{-3}$ L min^-1^. The breakthrough curves shown in Figure 6a) indicate that increasing the BC dose delays saturation and extends the operating time until system exhaustion, due to the greater availability of active sites for adsorption. This behaviour is also reflected in parameters reported in Table 4, which were obtained from fitting the Yan et al. model.

As shown in Table 4, the $q_{\gamma }$ values increased with higher adsorbent doses. Experimental results presented in Table 5 show that doubling the dose from 0.5 to 1.0 g L^-1^ resulted in an increase of only about 45% in the *t*_*b*_ and *V*_*b*_. When the dose was increased from 0.5 to 1.5 g L^-1^, i.e., tripling the amount of adsorbent, the *t*_*b*_ and *V*_*b*_ increased only approximately 2.2 times. This behaviour confirms that the relationship between adsorbent dose and ST performance is non-linear, indicating that increasing the BC dose does not result in a proportional gain in removal capacity. This is likely due to mass transfer limitations and/or restricted accessibility to pores and active sites, such as slow intraparticle diffusion or pore blockage, possibly because the mixture was not fully homogeneous. A dose of 0.5 g L^-1^ was selected as the most appropriate, ensuring satisfactory DCF removal performance.

**TABLE 5:** Characteristic parameters of ST system at different BC doses (0.5, 1.0, and 1.5 g L^-1^), different influent flow rates (6.94⋅ $10^{-4}$ , 1.39⋅ $10^{-3}$ and 2.78 ⋅ $10^{-3}$ L min^-1^) and for different adsorbent material (BC and BC-LAC) including breakthrough time (t_b_) and treated volume (V_b_).

Parameters	Adsorbent dose (g L^-1^)^d)^			Flow rate (L min^-1^)^e)^			Absorbent material^f)^	
	0.5	1.0	1.5	6.94 ⋅ $10^{-4}$	1.394 ⋅ $10^{-3}$	2.78 ⋅ $10^{-3}$	BC	BC-LAC
$t_{b}$ (min)	330	480	735	600	330	150	360	375
$V_{b}$ (L)	0.46	0.67	1.02	0.42	0.46	0.42	0.25	0.26

a Experiments conducted at a constant flow rate of 1.394 ⋅ $10^{-3}$ L min-1.

b Experiments conducted at constant adsorbent dose of 0.5 g L-1.

c Experiments conducted at a constant flow rate of 6.94 ⋅ $10^{-4}$ L min-1 and constant adsorbent dose of 0.5 g L-1.

##### 3.5.2.2 Effect of flow rate

After selecting the adsorbent dose, the influence of the flow rate on ST performance was evaluated. As illustrated in Figure 6b, the experimental breakthrough curves show that higher flow rates lead to a more pronounced increase in the relative concentration C/C_0_, resulting in faster system saturation. This behaviour is attributed to the reduced residence time of the solution, which limits the contact time between DCF and the active sites of BC.

Consequently, diffusion of the contaminant into the interior of the particles is restricted, reducing adsorption capacity, as previously reported in literature (Rocha et al., 2022). The breakthrough curves obtained at higher flow rates exhibit steeper slopes, indicating limitations in mass transfer within the adsorbent. The quantitative data derived from these curves are summarized in Table 5. Comparison of the results obtained at different flow rates shows that 6.94 ⋅ $10^{-4}$ L min^-1^ provided the longest operating time. Although a flow rate of 1.39 ⋅ $10^{-3}$ L min^-1^ resulted in a slightly higher *V*_*b*_ than 6.94 ⋅ $10^{-4}$ L min^-1^, this was achieved in a more gradual and stable manner at the lower flow rate. At 2.78 ⋅ $10^{-3}$ L min^-1^, system saturation occurred abruptly, with *C/C_0_* values rapidly approaching unity, indicating rapid adsorbent exhaustion. Overall, a flow rate of 6.94 ⋅ $10^{-4}$ L min^-1^ proved to be the most advantageous, significantly extending the system’s operational lifetime. Although 1.39 ⋅ $10^{-3}$ L min^-1^ allowed a marginally higher *V*_*b*_ in a shorter time, higher flow rates may compromise system efficiency and stability in real wastewater treatment applications due to accelerated saturation. Based on these conditions, a flow rate of 6.94 ⋅ $10^{-4}$ L min^-1^ was selected for subsequent experiments.

##### 3.5.2.3 Experiments with effluent and immobilized laccase

Figure 4c compares the performance of BC for DCF removal in buffered ultrapure water at pH 7.0 and in final effluent collected from WWTP. In both matrices, the saturation curve expressed by the *C/C_0_* ratio exhibits a sigmoidal profile, evolving towards values close to 1.0. It is observed that, in the effluent, saturation is reached slightly earlier than buffered water at pH 7.0.

The slightly lower performance in effluent can be attributed to several factors previously discussed in Experiments with effluent and immobilized laccase, in Section 3.5.1, namely pH variations that intensify electrostatic repulsion between DCF and BC, competition for adsorption sites due to the presence of other solutes, and partial pore blockage caused by organic matter. Nevertheless, the temporal shift between the two curves is relatively small, with a reduction of only 1.7, as the *t*_*b*_ was 600 min in buffered water at pH 7.0 and 360 min in effluent. This difference is particularly modest when compared with that observed in the FBC (Figure 5c) where the effect of matrix composition was much more pronounced.

The smoother discrepancy observed in the ST when compared with FBC case, can be explained by vigorous stirring which promotes complete mixing, reducing external film resistance and eliminating axial dispersion effects typically present in fixed bed system. As a result, the process is primarily governed by intraparticle kinetics within the BC and is therefore less sensitive to hydrodynamic variations between matrices. Nonetheless, the aforementioned factors account for the lower efficiency observed in effluent compared to buffered water.

Figure 6d presents the results using BC-LAC in the ST system. As shown in Table 5, the BC-LAC system exhibited slightly higher *t*_*b*_ and *V*_*b*_ than those observed for BC.

These results suggest that, at an initial stage, enzyme immobilization on BC may have provided a modest improvement in performance relative to non-functionalized BC, as evidenced by the marginal increase in *t*_*b*_ and *V*_*b*_. Nevertheless, at breakthrough, the *V*_*b*_ were very similar for both materials, namely 0.25 L and 0.26 L for BC and BC-LAC, respectively, as were the corresponding *t*_*b*_ of 360 min and 375 min. However, analysis of the complete breakthrough curves reveals that overall saturation (C/C_0_ ≈ 1) was reached at similar times for both materials, indicating that the initial performance gain observed with BC-LAC was not sustained over time.

The causes of these discrepancies were discussed in detail in Experiments with effluent and immobilized laccase, in Section 3.5.1, and are mainly associated with diffusional constraints imposed by enzyme immobilization. The initial improvement observed in the ST system was not detected in the FBC experiment. This difference can be explained by the distinct hydrodynamic and mass transfer conditions in each case. In the FBC, unidirectional flow and the development of concentration gradients along the bed render diffusional limitations, thereby suppressing potential benefits associated with the presence of LAC in the composite. In contrast, in the ST system, homogeneous mixing and the absence of longitudinal gradients, combined with the presence of two top openings that allow additional oxygen input and improved gas diffusion, favour enzymatic activity and slightly extend the *t*_*b*_. However, as the process progresses, the negative effects associated with LAC immobilization prevail, leading to saturation and reduced overall efficiency.

Direct comparison with literature remains limited due to the scarcity of studies reporting systems in semi-continuous mode under comparable experimental conditions, particularly those involving waste-derived BC and BC-LAC for DCF removal. Consequently, the results presented constitute a clear contribution beyond state of the art.

In summary, both BC and BC-LAC exhibit good performance in ST system, with BC-LAC providing slightly higher *t*_*b*_ and *V*_*b*_ values under suitable operating conditions. Nonetheless, this effect is limited and must be weighed against the additional costs associated with enzymatic immobilization. Thus, despite the potential of BC-LAC, BC remains the more robust option in terms of sustained performance.

## 4 Conclusion

This work addresses the application of BC for wastewater treatment, highlighting the potential to valorise industrial residues by converting them into adsorbents with suitable textural properties. Microwave-assisted pyrolysis proved to be an efficient route for BC production, yielding a relatively high specific surface area and well-developed porous structure, key characteristics for adsorption processes.

With respect to the preliminary batch experiments, demonstrated that BC can remove DCF efficiently, achieving removal values exceeding 80% under the tested conditions, in accordance with the removal targets established by Directive (EU) 2024/3019 (EUR-Lex, 2024). Nevertheless, some limitations were observed, associated with unfavourable electrostatic interactions between DCF and adsorbent surface. The adsorption kinetics data were well described by pseudo-second-order model, while equilibrium data were satisfactorily fitted by Langmuir-Freundlich model, suggesting predominantly monolayer formation on energetically heterogeneous sites.

Building on these batch results, the performance of BC was further evaluated under semi-continuous operating conditions (FBC and ST systems). Under the most favourable conditions, FBC and ST experiments exhibited distinct but still noteworthy performances, with the Yan et al. model providing an excellent description of the breakthrough curves. In FBC, the *t*_*b*_ occurred much faster for effluent than for buffered water at pH 7.0 (≈72.7 min vs. 596.8 min), corresponding to a *V*_*b*_ of approximately eight times lower. In contrast, in ST system, saturation with effluent was much less pronounced, with a *t*_*b*_ only 1.7 times lower than that observed in buffered water at pH 7.0, indicating longer contact times and more effective diffusion in the liquid medium.

The physical immobilization of laccase onto BC (BC-LAC) showed low efficiency, resulting in a limited amount of enzyme effectively bound to the support. Moreover, rather than enhancing performance in the FBC, diffusional limitations and partial pore blockage were observed, leading to an overall performance inferior to that with non-functionalized material. In fact, the FBC operated with BC showed higher removal efficiency, treating a volume of effluent five times higher than that achieved with BC-LAC. In contrast, in the ST system, BC-LAC breakthrough curve revealed a slight initial improvement in DCF removal compared with BC, which can be attributed to favourable hydrodynamic conditions and the presence of additional oxygen inputs, which may have contributed to improved oxygen availability and mass transfer for enzymatic activity. Nevertheless, under the operating conditions, both *V*_*b*_ (0.25 and 0.26 L for BC and BC-LAC, respectively), as well as the corresponding *t*_*b*_ (360 and 375 min for BC and BC-LAC, respectively) were similar for both materials. These results suggest that although BC-LAC presents potential, its application requires suitable operating conditions, including adequate oxygen availability and the need to optimize immobilization methods, taking associated costs into account.

This study expands current knowledge on the application of enzyme functionalized and non-functionalized waste-derived BC in semi-continuous systems for DCF removal. Overall, this study highlights the potential of waste-derived biochar as an effective adsorbent, within circular strategies for green chemical upgrading and sustainable water treatment. However, the integration of adsorption with biocatalysis did not enhanced cost-effectiveness under the evaluated conditions. Therefore, continuous research will be essential to overcome the identified challenges and to consolidate the potential of these materials as sustainable and economically viable alternatives to meet increasing regulatory and environmental demands.

## Data Availability

The original contributions presented in the study are included in the article/Supplementary Material, further inquiries can be directed to the corresponding author.
