# Supplementary material for: Semi-continuous adsorption-biocatalysis systems using waste-derived biochar functionalized with laccase for diclofenac removal from wastewater
Source: Front Chem. 2026 May 25;14:1814099. doi: 10.3389/fchem.2026.1814099 (PMC13243280; doi:10.3389/fchem.2026.1814099)
Supplement: Supplementary file 1 [file Supplementaryfile1.docx]

Supplementary Material

Semi-continuous adsorption-biocatalysis systems using waste-derived biochar functionalized with laccase for diclofenac removal from wastewater

Rita Gouveia^1^, Ângela Almeida^2^, Érika M. L. Sousa^2^, María V. Gil^3^, Francisco Freitas^4^, Vânia Calisto^2*^

^1^Department of Chemistry, University of Aveiro, Campus de Santiago, 3810-193 Aveiro, Portugal

^2^Department of Chemistry and CESAM, University of Aveiro, Campus de Santiago, 3810-193 Aveiro, Portugal

^3^Instituto de Ciencia y Tecnología del Carbono (INCAR), CSIC, Francisco Pintado Fe 26, 33011 Oviedo, Spain

^4^Department of Chemistry and CICECO, University of Aveiro, Campus de Santiago, 3810-193 Aveiro, Portugal

*** Correspondence:**Vânia Calisto
vania.calisto@ua.pt

# Effluent characterization

The physicochemical parameters of the effluent were characterized using a portable multiparameter instrument (HI98494, Hanna Instruments), which measured pH, electrical conductivity, and temperature. Total organic carbon (TOC) was determined using a TOC analyzer (Shimadzu, model TOC-V CPH, with SSM-5000A module). The calibration curve was prepared using potassium hydrogen phthalate (KHC_8_H_4_O_4_) standard solutions in ultrapure water, within a concentration range of 0.1 to 10.0 mg L^-1^, obtained by dilution of a 1000 mg L^-1^ stock solution. Calibration validation was performed using a freshly prepared 5.0 mg L^-1^ KHC_8_H_4_O_4_ standard solution. Prior to analysis, wastewater samples were filtered through 0.22 µm polyvinylidene fluoride (PVDF, Whatman) syringe filters, acidified with 2% (v/v) 2 mol L^-1^ HCl, and sealed with Parafilm M^®^ until organic carbon determination. The results of the effluent characterization are presented in Table S1.

The wastewater properties were similar in both sampling campaigns.

# Detail experimental procedures for characterization methods

Textural properties were determined by N_2_ adsorption–desorption isotherms, using a Micromeritics Gemini VII 2380 analyzer (USA). Prior to analysis, the samples were degassed at 120 °C overnight. The isotherms were recorded at the temperature of liquid N₂ (−196 °C). S_BET_ was calculated using the BET equation (Dubinin, 1975) over the relative pressure (P/P₀) range of 0.01–0.10. The micropore volume (*W*₀) was obtained using the Dubinin–Astakhov equation (Dubinin, 1975). The total pore volume (*V*_p_) was estimated from the amount of N₂ adsorbed at P/P₀ = 0.99. The mean pore diameter (*D*) was then determined from V_p_ and S_BET_ , as described by *Sousa et al.* (2022).

SEM was used to analyse the surface morphology of the produced materials. Images were acquired using a Hitachi S4100 instrument (Japan) at magnifications of 500× and 6000×.

The PZC of BC was determined using the pH-drift method. Aqueous 0.1 M NaCl solutions were prepared with initial pH values (pH_i_) ranging from 3 to 11, adjusted with 0.01 M or 0.1 M HCl or NaOH solutions. For each pH value, 40 mL of solution were added to 20.0 mg of BC (corresponding to a BC dose of 500 mg L⁻¹) in polypropylene tubes, and the suspensions were agitated on a rotary shaker for 24 h at 25 °C. After incubation, the suspensions were filtered and the final pH (pH_f_) was measured. The PZC was obtained by plotting the pH variation (ΔpH = pH_f_ − pH_i_) as a function of pH_i_; thus, the PZC of BC corresponds to the intersection of the curve with the *x*-axis (ΔpH = 0).

Enzymatic activity was determined following an adaptation of the method of Ander and Messner (1998), using ABTS as the substrate. This allowed the assessment of the activity of free LAC (LAC_F_) in the washing leachates (i.e., LAC in the wash filtrates) and immobilized LAC (LAC_I_) in the composites. All analyses were performed in triplicate and results are reported as the mean values. Briefly, to evaluate the activity of LAC_I_, 2.00 mg of the composite were suspended in an ABTS solution (0.4 mM in 0.1 M acetate buffer, pH 3.5) and subjected to agitation at 350 rpm and 40 ºC for 8 min. After the reaction, the samples were filtered through syringe filters (0.2 µm, PTFE, Labfil). From the filtrate, 300 µL were transferred to a microplate, and the absorbance was monitored at 420 nm in 96-well microplate using a UV–Vis microplate spectrophotometer (SpectraMax 190, Molecular Devices). Thus, based on the slope of the initial linear region of the absorbance-time curve, the catalytic activity (U mL^-1^) can be calculated, where one unit (U) corresponds to the amount of enzyme require to oxide 1 µmol of ABTS per minute. The calculation of LAC_I_ enzymatic activity (AE_LACI_) can be described by equation 1:

$$\begin{aligned} {AE}_{LACI}=\frac{\frac{\Delta Abs}{min}\cdot V_{reaction}\cdot{10}^{6}}{\varepsilon_{ABTS}\cdot m_{composite}} \#\left( 1 \right) \end{aligned}$$

Where $\frac{\Delta Abs}{min}$ corresponds to the slope of the initial linear region of the kinetic curve, $\varepsilon_{ABTS}$ (L mol^-1^ cm ^-1^) is the molar absorptivity coefficient of ABTS ($\varepsilon_{420}=$ 3.6 ∙${10}^{4}$ M^‑1^cm^‑1^), $V_{reaction}$ (mL) is the total reaction volume, $m_{composite}$ (g) is the mass of the composite and ${10}^{6}$ is the conversion factor from mol L^-1^ to µmol L^-1^.

# Quantification of DCF by chromatographic analysis

DCF quantification was performed by high-performance liquid chromatography with ultraviolet detection (HPLC-UV). Analyses were carried out using a Waters Alliance 2695 system coupled to a Waters 2487 dual-absorbance detector, operated with Empower 3 software. Chromatographic separation was achieved on a reversed-phase ACE^®^ C18-PFP column (150 mm × 4.6 mm of internal diameter, 5 µm particle size), equipped with an ACE^®^ 5 C18 guard column (4.6 mm), maintained at 25 ºC.

The mobile phase consisted of 65% acetonitrile and 35% ultrapure water acidified with phosphoric acid (≈ pH 2.6), at a flow rate of 0.8 mL min^-1^. Both mobile phase solvents were previously filtered through 0.22 µm polyamide membrane filter (Whatman). Prior to analysis, all samples and DCF standard solution were filtered using 0.22 µm PVDF syringe filters (Whatman). The injection volume was 20 µL, and DCF detection was performed at 220 nm.

DCF stock solution at 10 mg L^-1^ were prepared for each matrix investigated (ultrapure water, 1mM acetate buffer at pH 7.0, or wastewater), as required for the construction of calibration curves, and subsequently diluted to obtain a concentration range of 0.1-5 mg L^-1^. Calibration curves were fitted by linear regression using the least-squares method. The limits of detection (LOD) and quantification (LOQ) were determined according to the equations proposed by Miller and Miller (Gegenschatz et al., 2022).

# Kinetic and isotherm models

The study of different kinetic models is essential to describe experimental data and to assist in identifying the factors that control adsorption in an adsorbent-adsorbate system. Adsorption kinetics therefore describes the rate at which the adsorbate is removed from liquid phase and fixed onto the adsorbent over time, being intrinsically related to mass transfer phenomena. The analysis is based on the temporal variation of the solute concentration in the aqueous phase, $C$ (mg L^‑1^), or on the adsorption capacity, $q$ (mg g^-1^), defined as the amount of solute adsorbed per unit mass of adsorbent (Ferreira, 2017). Among the most relevant parameters, the equilibrium adsorption capacity, $q_{e}$ (mg g^-1^), stands out, representing the amount of absorbate retained on the adsorbent when system reaches equilibrium. Its value is determined using Equation 2:

$$\begin{aligned} q_{e}=\frac{\left( C_{0}-C_{e} \right)\cdot V}{m_{A}} \#\left( 2 \right) \end{aligned}$$

Where $C_{0}$ (mg g^-1^) and $C_{e}$(mg L^-1^) represent the initial and equilibrium concentrations of the adsorbate in solution, respectively $V$ (L) is the solution volume and $m_{A}$ (g) is the mass adsorbent.

The fundamental equation of the pseudo-first-order kinetic model is expressed by Equation 3 (Ribas et al., 2014):

$$\begin{aligned} q_{t}=q_{e}\cdot\left( 1-e^{-k_{1}\cdot t} \right) \#\left( 3 \right) \end{aligned}$$

Where $q_{t}$ (mg g^-1^) is the adsorption capacity at time t, $k_{1}$ (min^-1^) is the pseudo-first-order kinetic rate constant and $t$ (min) is time.

The pseudo-second-order kinetic model, proposed by Ho *et al.* (Ferreira, 2017) is widely used to describe adsorption processes in liquid-solid systems. The equation governing this model is expressed as (Ferreira, 2017):

$$\begin{aligned} q_{t}=\frac{k_{2}\cdot q_{e}^{2}\cdot t}{1+k_{2}\cdot q_{e}\cdot t} \#\left( 4 \right) \end{aligned}$$

Where $k_{2}$ (g mg^-1^ min^-1^) is the pseudo-second-order adsorption rate constant.

The Elovich equation, represented by Equation 5, is frequently used to describe gas adsorption on heterogeneous solids, as well as the adsorption of contaminants from aqueous solution (Nascimento et al., 2014):

$$\begin{aligned} q_{t}=\beta\cdot\ln\left( \alpha\cdot\beta\right)+\beta\cdot\ln\left( t \right) \#\left( 5 \right) \end{aligned}$$

The coefficient $\alpha$ (mg g^-1^ min^-1^) and $\beta$ (g mg^-1^) represent the initial adsorption rate and the desorption coefficient, respectively.

The intraparticle diffusion model is particularly relevant for porous adsorbent, as it describes the transport of adsorbate molecules or ions into the pore of the adsorbent. In some cases, this diffusion may be the rate-determining step of the adsorption process, thus becoming a critical aspect of adsorption kinetics (Sen Gupta and Bhattacharyya, 2011). This relationship is expressed by Equation 6:

$$\begin{aligned} \ln\left( 1-\frac{q_{t}}{q_{e}} \right)=-\frac{\pi^{2}\cdot D_{c}}{r^{2}}\cdot t+\ln\left( \frac{6}{\pi^{2}} \right) \#\left( 6 \right) \end{aligned}$$

Where $D_{c}$ (cm^2^ s^-1^) represents the intracrystalline diffusivity and $r$ (cm) is the radius of the adsorbent particles. This model assumes that the rate-limiting step of the adsorption process is solute diffusion within the particle, as this step is considered much slower than the others. Surface adsorption can be regarded as instantaneous (Qiu et al., 2009), allowing the process to be described by a single step, as expressed by Equation 7:

$$\begin{aligned} q_{t}=k_{in}\cdot t^{1/2}+C_{ID} \#\left( 7 \right) \end{aligned}$$

Where $k_{in}$ (mg g^-1^ min^-1/2^) is the adsorption rate constant and $C_{ID}$ (mg g^-1^) is the intraparticle diffusion constant, which also provides information on the thickness of the boundary layer (the liquid film adjacent to the adsorbent surface that offers additional resistance to mass transfer).

Isotherms are used to evaluate the adsorption capacity of an adsorbent toward a given molecule and to estimate the selectivity of different adsorbates for a specific adsorbent. Several mathematical models describe adsorption isotherms, each based on different assumptions regarding the nature of the adsorbent surface and the interactions between adsorbed molecules (Ferreira, 2017).

The Langmuir model, proposed by Langmuir (Nascimento et al., 2014), was the first to provide a theoretical basis for adsorption, initially developed to describe gas adsorption on solid surfaces and later applied to liquid systems. The equation is given by:

$$\begin{aligned} q_{e}=\frac{q_{max,L}\cdot K_{L}\cdot C_{e}}{1+K_{L}\cdot C_{e}} \#\left( 8 \right) \end{aligned}$$

Where $q_{max,L}$ (mg g^-1^) is the maximum Langmuir adsorption capacity and $K_{L}$(L mg^-1^) is the Langmuir equilibrium constant.

The Freundlich isotherm is empirical and describes heterogeneous adsorption on surfaces with different types of active sites, allowing for multilayer formation. The total adsorption results from the sum of individual adsorption events at different sites with varying affinities, where higher-energy sites are occupied first, followed by an exponential decrease in adsorption energy (Nascimento et al., 2014). The Freundlich model is described by Equation 9:

$$\begin{aligned} q_{e}=K_{F}\cdot C_{e}^{1/{NF}} \#\left( 9 \right) \end{aligned}$$

Where $K_{F}$ (mg g^-1^ (mg L^-1^)^-1/NF^) is the Freundlich constant and $NF$ is the Freundlich heterogeneity parameter related to the degree of non-linearity of the equation. Values of 0 < $NF$ < 1 indicate high adsorption affinity (favorable adsorption), whereas $NF$ > 1 indicates unfavorable adsorption, and $NF$ = 1 corresponds to linear adsorption with identical adsorption energies for all sites (Nascimento et al., 2014; Ferreira, 2017).

The Langmuir-Freundlich model describes heterogeneous adsorption systems and overcomes the limitations of the original models. The model is represented by Equation 10 (Ferreira, 2017):

$$\begin{aligned} q_{e}=\frac{{q_{max,LF}\cdot K}_{LF}\cdot C_{e}^{1/{NLF}}}{1+K_{LF}\cdot C_{e}^{1/{NLF}}} \#\left( 10 \right) \end{aligned}$$

Where $q_{max,LF}$ (mg g^-1^) represents the maximum Langmuir-Freundlich adsorption capacity, $K_{LF}$ (mg g^-1^ (mg L^-1^)^-1/NLF^) is the Langmuir-Freundlich affinity constant and $NLF$ is the heterogeneity (non-linearity) parameters (Ferreira, 2017).

The Linear-Langmuir model is a derivation of the Langmuir model obtained by combining it with a linear model, resulting in an expression frequently used due to its ease of linearization, which allows the estimation of adsorption parameters from experimental data (Murphy et al., 2023). The general form of this isotherm is expressed as:

$$\begin{aligned} q_{e}=m_{LL}\cdot C_{e}+\frac{q_{max,LL}\cdot K_{LL}\cdot C_{e}}{1+K_{LL}\cdot C_{e}} \#\left( 11 \right) \end{aligned}$$

Where $m_{LL}$ (mg g^-1^ (L mg^-1^)) is the coefficient of the linear component, $q_{max,LL}$ (mg g^-1^) represents the maximum Linear-Langmuir adsorption capacity and $K_{LL}$ (L mg^-1^) is the Langmuir affinity constant.

# Schematic figures and real images of the experimental systems

The FBC experimental setup employed for DCF adsorption using BC is illustrated in a photograph of the system in operation is presented in Figure S1. The configuration used for BC-LAC, Figure S2 was identical to that described above, within the addition of continuous compressed air supply to the feed solution to ensure oxygen availability during enzymatic operation.

The ST configuration employed for DCF adsorption onto BC is presented in Figure S3 with a photographic representation of the operation system. In the case of BC-LAC, Figure S4, the excerpt for the incorporation of continuous compressed air bubbling into the reactor to maintain adequate oxygen availability required for LAC catalytic performance.

References

Dubinin, M. M. (1975). “Physical Adsorption of Gases and Vapors in Micropores,” in *Progress in Surface and Membrane Science*, eds. D. A. Cadenhead, J. F. Danielli, and M. D. Rosenberg (Elsevier), 1–70. doi: 10.1016/B978-0-12-571809-7.50006-1

Ferreira, C. I. A. (2017). Production and application of biowaste-based adsorbents for the removal of fish anaesthetics in recirculating aquaculture systems. Aveiro: Universidade de Aveiro. Available at: https://ria.ua.pt/handle/10773/18042 (Accessed June 9, 2025).

Gegenschatz, S. A., Chiappini, F. A., Teglia, C. M., Muñoz de la Peña, A., and Goicoechea, H. C. (2022). Binding the gap between experiments, statistics, and method comparison: A tutorial for computing limits of detection and quantification in univariate calibration for complex samples. *Analytica Chimica Acta* 1209, 339342. doi: 10.1016/j.aca.2021.339342

Murphy, O. P., Vashishtha, M., Palanisamy, P., and Kumar, K. V. (2023). A Review on the Adsorption Isotherms and Design Calculations for the Optimization of Adsorbent Mass and Contact Time. *ACS Omega* 8, 17407–17430. doi: 10.1021/acsomega.2c08155

Nascimento, R. F. do, Lima, A. C. A. de, Vidal, C. B., Melo, D. de Q., and Raulino, G. S. C. (2014). Adsorção: aspectos teóricos e aplicações ambientais. 1, 258.

Qiu, H., Lv, L., Pan, B., Zhang, Q., Zhang, W., and Zhang, Q. (2009). Critical review in adsorption kinetic models. *J. Zhejiang Univ. Sci. A* 10, 716–724. doi: 10.1631/jzus.A0820524

Ribas, M. C., Adebayo, M. A., Prola, L. D. T., Lima, E. C., Cataluña, R., Feris, L. A., et al. (2014). Comparison of a homemade cocoa shell activated carbon with commercial activated carbon for the removal of reactive violet 5 dye from aqueous solutions. *Chemical Engineering Journal* 248, 315–326. doi: 10.1016/j.cej.2014.03.054

Sen Gupta, S., and Bhattacharyya, K. G. (2011). Kinetics of adsorption of metal ions on inorganic materials: A review. *Advances in Colloid and Interface Science* 162, 39–58. doi: 10.1016/j.cis.2010.12.004

Sousa, É. M. L., Otero, M., Rocha, L. S., Gil, M. V., Ferreira, P., Esteves, V. I., et al. (2022). Multivariable optimization of activated carbon production from microwave pyrolysis of brewery wastes - Application in the removal of antibiotics from water. *Journal of Hazardous Materials* 431, 128556. doi: 10.1016/j.jhazmat.2022.128556

Verlicchi, P., Al Aukidy, M., and Zambello, E. (2012). Occurrence of pharmaceutical compounds in urban wastewater: Removal, mass load and environmental risk after a secondary treatment—A review. *Science of The Total Environment* 429, 123–155. doi: 10.1016/j.scitotenv.2012.04.028

**Table S1 –** Effluent properties measured in June and July 2025.

| Parameters | Sampling date | |
| --- | --- | --- |
|  | June 2025 | July 2025 |
| pH | 8.02 | - |
| Conductivity (µS cm^-1^) | 3607 | - |
| Resistivity (MΩ cm^-1^) | 0.0003 | 0.0003 |
| Total Dissolved Solids (mg L^-1^) | 1804 | 1960 |
| Salinity (PSU) | 1.91 | 2.09 |
| Temperature (ºC) | 16.79 | 11.03 |
| Total Organic Carbon (mg L^-1^)* | 15.87 ± 0.08 | 15.09 ± 0.07 |

*Parameter measured using TOC analyzer. All remaining parameters were measured using the Hanna HI9894 multiparameter probe.

**Figure S1 -** Real photograph of the FBC system in operation during DCF adsorption experiments.

**Figure S2 -** Diagram illustrating the components of the FBC system, including feed tank, peristaltic pump, CHROMAFLEX® glass column packed with BC-LAC, thermostatic recirculating bath, and sample collection unit. (Created in BioRender. Gouveia, R. (2026) https://BioRender.com/fje4ekh).

**Figure S3** - Photograph of the ST system in operation during DCF adsorption experiments.

**Figure S4 -** Diagram illustrating the components of the stirred tank, including feed tank, peristaltic pump, tank with BC-LAC, thermostatic recirculating bath, and sample collection unit. (Created in BioRender. Gouveia, R. (2026) <https://BioRender.com/nablmud>).
