# Supplementary figures and images for: Semi-continuous adsorption-biocatalysis systems using waste-derived biochar functionalized with laccase for diclofenac removal from wastewater
Source: Front Chem. 2026 May 25;14:1814099. doi: 10.3389/fchem.2026.1814099 (PMC13243280; doi:10.3389/fchem.2026.1814099)

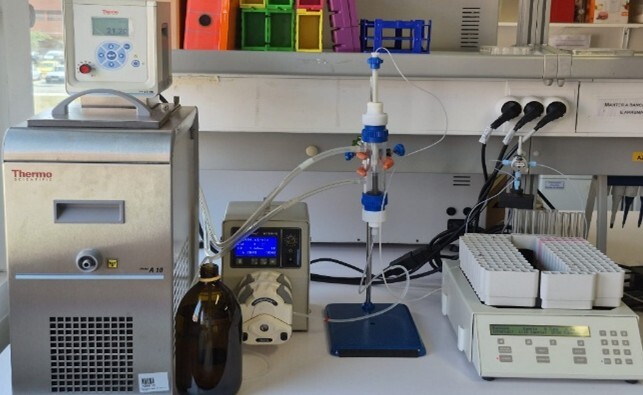

Supplement: Supplementary file 2 [file Supplementaryfile2.zip › Figuras SM/Figure S1.jpg]

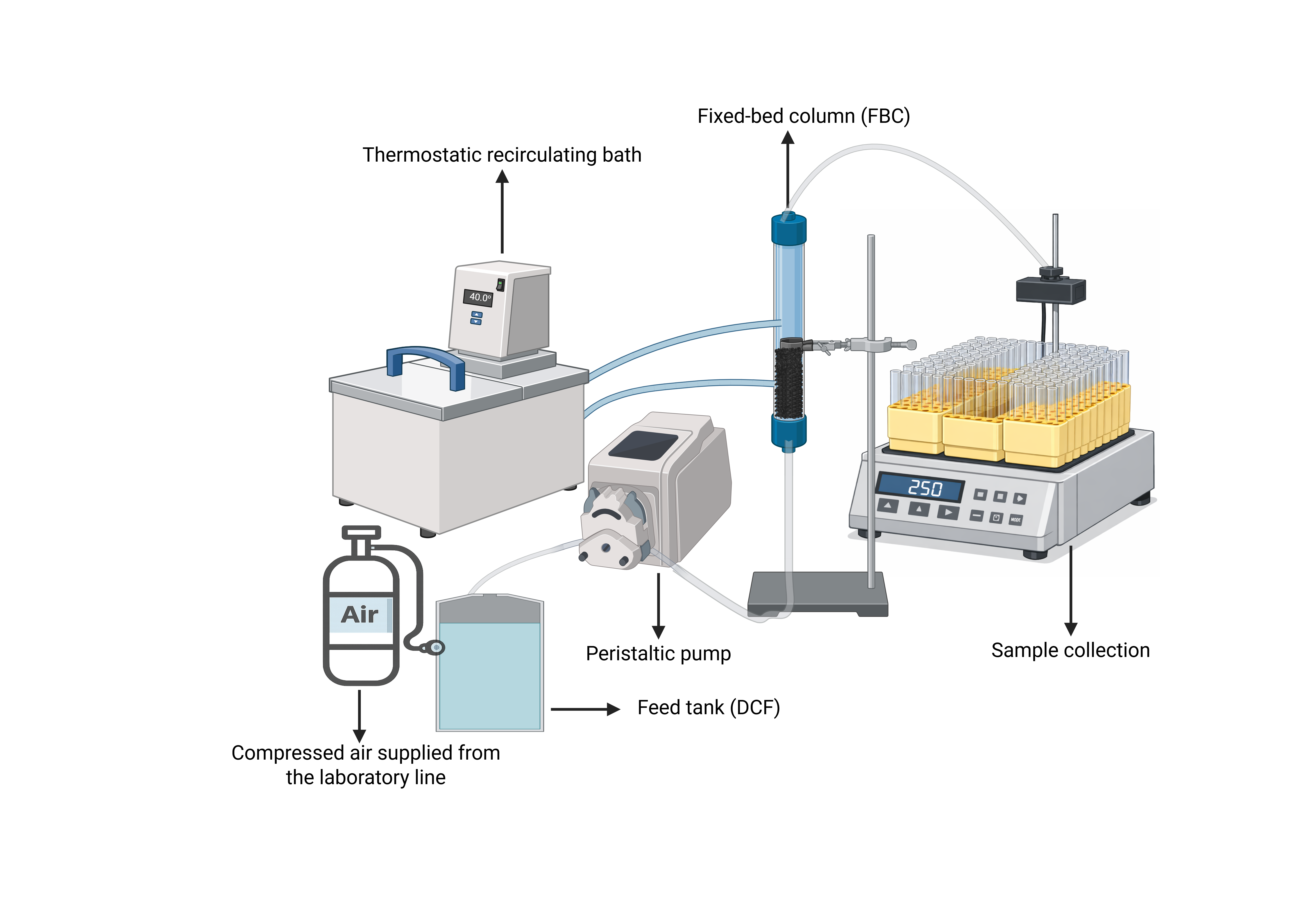

Supplement: Supplementary file 2 [file Supplementaryfile2.zip › Figuras SM/Figure S2.png]

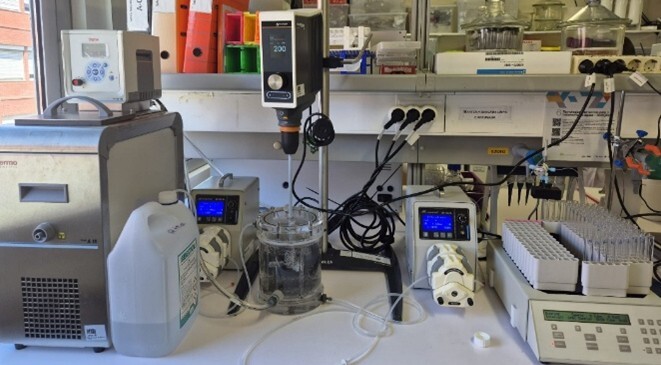

Supplement: Supplementary file 2 [file Supplementaryfile2.zip › Figuras SM/Figure S3.jpg]

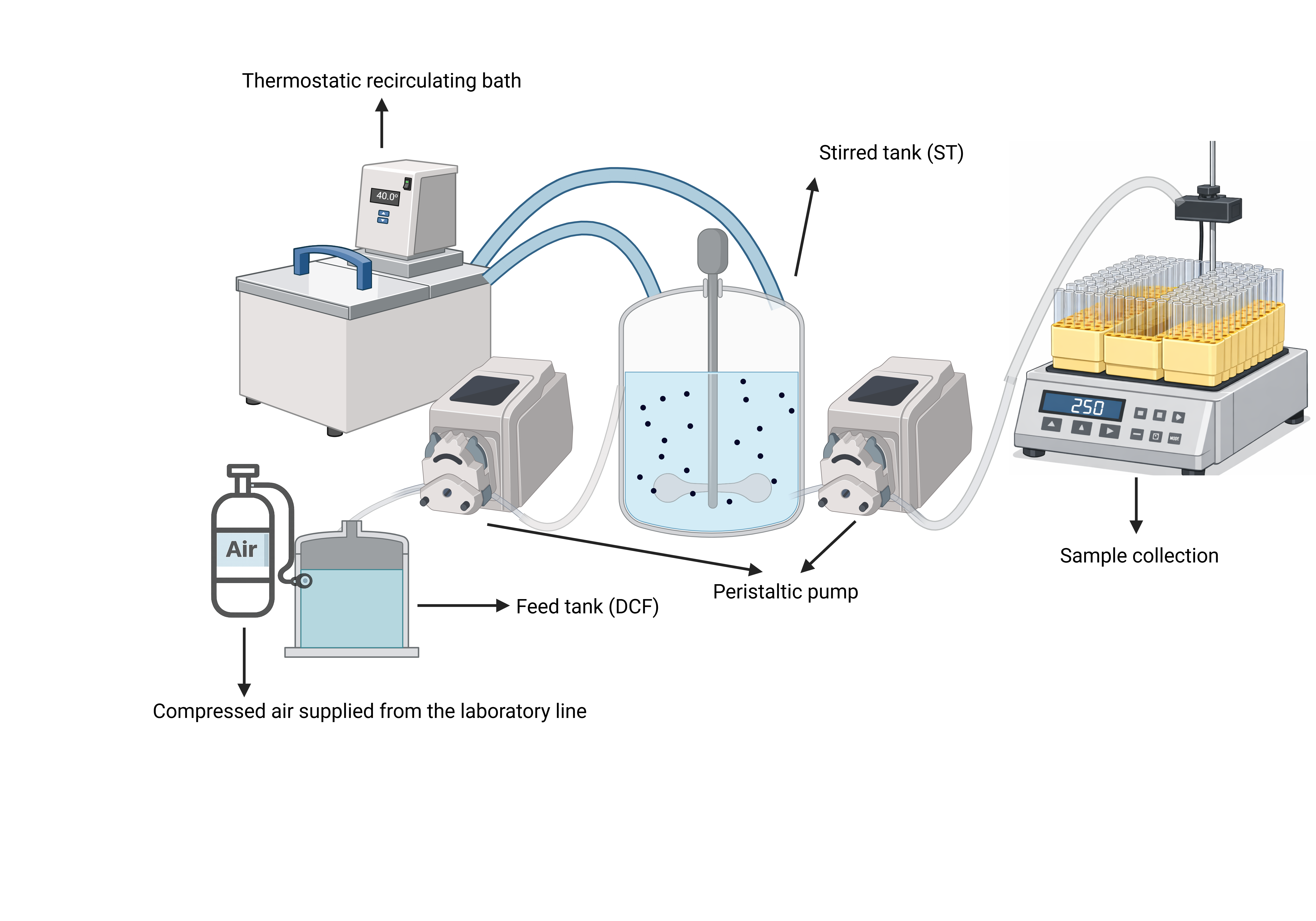

Supplement: Supplementary file 2 [file Supplementaryfile2.zip › Figuras SM/Figure S4.png]
